# Supplementary material for: A whole-animal phenotypic drug screen identifies suppressors of atherogenic lipoproteins
Source: eLife. 2026 Aug 27;14:RP105314. doi: 10.7554/eLife.105314 (PMC13521566; doi:10.7554/eLife.105314)
Supplement: Supplementary file 2 [file elife-105314-code1.zip › JHDL_primary_result/Thiethylperazine malate/Thiethylperazine malate_JHDL_34_025_030_2020_01_31_11_18.pdf]

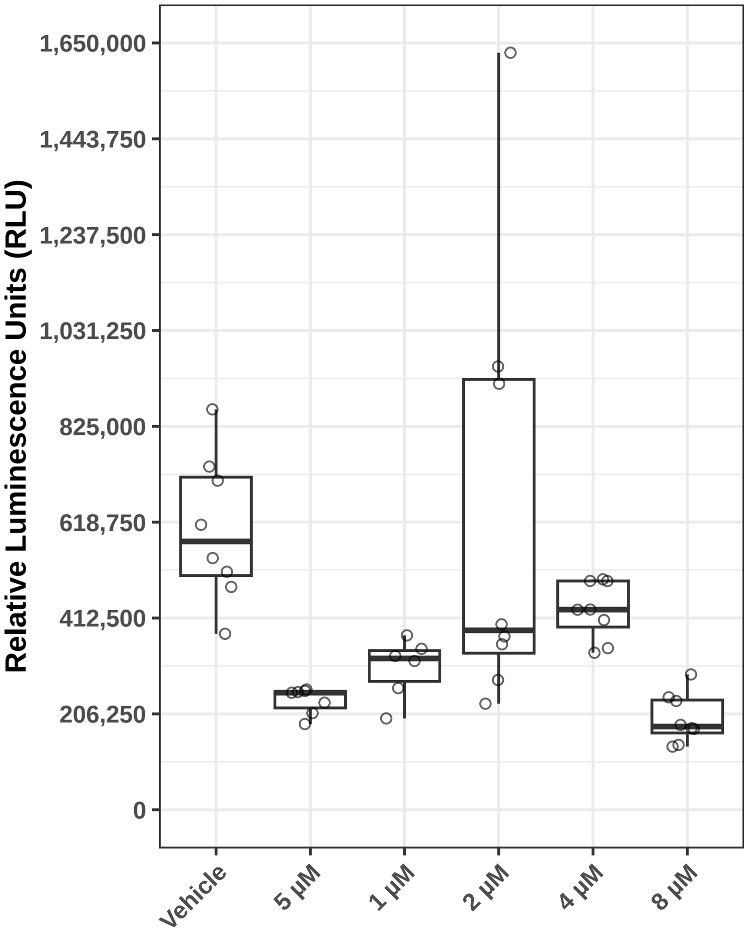
Relative Luminescence Units (RLU)
1,650,000
1,443,750
1,237,500
1,031,250
825,000
618,750
412,500
206,250
0
Vehicle
5 μM
1 μM
2 μM
4 μM
8 μM
